# Termite Ecology in the First Two Decades of the 21st Century: A Review of Reviews

**DOI:** 10.3390/insects10030060

**Published:** 2019-02-26

**Authors:** David E. Bignell

**Affiliations:** School of Biological and Chemical Sciences, Queen Mary University of London, Mile End Road, London E1 4NS, UK; d.bignell@qmul.ac.uk; Tel.: +44-1737-218047

**Keywords:** termite literature, Web of Science^TM^, ecosystem services, assemblage data, termite constructions, pedogenesis

## Abstract

Termite ecology came of age in 1978 with the seminal review of Wood and Sands which by considering the quantitative contributions made by termites to the carbon cycle at the landscape level concluded that they were major players in tropical ecosystems. Subsequent field work in the succeeding two decades was summarised in 2000 by Bignell and Eggleton, the most recent review which attempted to cover the entire topic in detail, which included 188 listed references and has been extensively cited for almost 20 years. Subsequent summaries more narrowly defined or in some cases more superficial are listed in the bibliography. In this overview, the main and subsidiary headings in Bignell and Eggleton are revisited and reclassified in the light of 186 selected articles added to the relevant literature since 2000, and some earlier work. While the literature on termite ecology remains buoyant, it has declined relative to publications on other aspects of termite biology. Overall, the thesis that termites have a major impact on, and are major indicators of soil health and landscape integrity in the tropics and sub-tropics is maintained, but the drivers of local diversity, abundance and biomass remain complex, with many biographical, edaphic and optimum sampling issues not completely resolved. The large increase in diversity and abundance data from Neotropical biomes can also be noted.

## 1. Introduction

Termites have a significant impact on pedogenesis, soil properties and soil functions over large areas of the tropics and sub-tropics, although the effect diminishes somewhat at higher latitudes [1]. The impact is variable with temperature, rainfall, altitude, seasonality, latitude, longitude and parent geology; but is generally the consequence of high abundance and biomass, their ability to shred, digest and/or process complex organic materials combined with the habit of creating extensive gallery systems in soil and the use of excavated mineral material to build mound-nests both above- and below-ground and runways and/or sheeting above ground. This manipulation affects the mineral components of soils in addition to the organic components both physically and chemically. Broadly, soils which are well populated by termites are better drained, more stable and likely to have a higher retained organic content than counterpart soils which are depauperate, either for natural reasons or because of anthropogenic land-use change. Additionally, termites can efficiently digest a wide range of types of organic detritus, from (in different taxa) freshly dead wood and dried grass through to highly humified organic-rich whole soil. This arises from a number of evolved mutualisms with microbes and appears to give them a role in decomposition processes out of proportion to their diversity and taxonomic identity as a single invertebrate taxon (currently an infraorder or epifamily), though perhaps not to their biomass. There is also evidence of a role in the N-cycle, especially symbiotic nitrogen fixation, which may be the primary contribution of some of the associated microbes, rather than the degradation of lignocellulose, and may result in enhanced nitrogen availability for local ecosystems.

All termites are eusocial; that is, the ability to reproduce can be suppressed in individuals (usually the great majority), which then reversibly or irreversibly specialize behaviourally and morphologically for other activities such as defence, foraging, construction or nurturing of the young. The result is a colony, ranging (in different cases) from a few individuals to many millions, where the members often (though not invariably) have a relatively high degree of relatedness to one another, as well as an absolute dependence on the continuation of the colony and its social order. Extant sub-social termites are not known. The impact of termites in soils is not a direct consequence of their social organization (for example, earthworms can have a comparable or greater impact without being eusocial), but inasmuch as the mutualisms with microbes have developed in a social context, sociality has been strongly selected in termite evolution and remains essential for their continued existence and niche exploitations [2].

## 2. Methodology

The notional purpose of this chapter is to provide an overview of termite ecology, but the subject has become so voluminous (Web of Science^TM^ identifies more than 500 published articles since 1970 using the keywords “termite” and “ecology”) that it is more manageable to focus on the first two decades of the 21st century and convenient as an approach to chart the directions the literature has taken since 2000. At the turn of the century, Kluwer published *Termites: Evolution, Sociality, Symbioses, Ecology* [3], the first comprehensive English language research compendium for 30 years, in which four chapters were allocated to the broader environmental aspects of termite biology: population dynamics [4], termites and soil properties [5] and global impacts [6], in addition to Bignell and Eggleton [7] who addressed the character and role of termite assemblages in ecosystems, landscapes and biomes. This last chapter, widely cited, encompasses what is generally considered to be the broad sweep of termite ecology and it, therefore, seems appropriate to consider how the views and assumptions it contains have changed and developed over the years. Current literature balance between different aspects of termite biology over the period 2000–2018 are summarised in Table 1.

Table 2 shows the main headings and subheadings set out by Bignell and Eggleton [7] and, for each, the number of citations included in the corresponding paragraphs. In addition, the number of articles published in the period 2000–2018 (as determined by a search in Web of Science^TM^) is given for each existing main heading and subheading, together with new headings and subheadings which are suggested by the development of termite ecology since 2000. Note that articles published before 2000 can be allocated to more than one heading or subheading, in which case the totals given at the foot of the table reflect the number of allocations as well as the number of articles. “New articles” include some published before 2000 and not included in [7] but which can now be considered as having continuing relevance. Though complex and somewhat awkward, this analysis gives some indications of the development and structure of the contemporary literature. The literature review was completed on 30 November 2018.

The bibliography given below mostly consists of articles which can be considered in whole or in part as reviews, together with a small number from the primary literature regarded as pivotal.

## 3. Literature Dynamics

Literature dynamics between 2000 and 2018 are shown in Table 1, based on a search of the Web of Science^TM^ database using either the single keyword “termite” or “termite” and one other term. This is an updated and simplified extension of Figure 6.1 in [8]; of the 6722 articles retrieved concerned with termites, 6.5% explicitly concerned termite ecology. Annual publication outputs on ecology sensu stricto (not shown) have declined sharply from a peak in 2007, while papers on symbiont biology and the digestion of cellulose and lignin have either increased or been sustained. Genomic, proteomic, metagenomic and metabolomic studies remain a small proportion, but are increasing. The trends may be attributed to growing interest in the role of termites as models for the degradation of lignocellulose, roughly twice as efficient in the insect as in a mammalian ruminant [9] with biofuel as a possible end-product if the process can be up-scaled industrially [10]. Further interest in termites stems from their usefulness as models of social organisation, the genetics of altruism and conflict, and of anti-microbial hygiene. These topics are beyond the scope of this article but may be followed up from chapters in Bignell et al. [11]

## 4. Ecology of Termites in Four Paragraphs

Environmentally, the main impact of termites is their role as soil ecosystem engineers in the tropics and subtropics, a function matched on the global scale only by earthworms and ants [1,12,13]. The net effect is to condition soil (to facilitate drainage and intimately mix its organic and inorganic constituents, preserving long term nutrient pools) and drive the decomposition arm of the carbon cycle [14]. It should be noted that decomposition does not simply mineralise dead tissue as CO_2_ (and to some extent as CH_4_); it also includes nitrification and the creation of carbon pools in soils, both of which promote and sustain fertility. There is also a shredding role, overlooked in many assessments of termite ecological importance [15,16]. Evidence that termite populations promote crop yields and can help to rehabilitate degraded soils and biologically impoverished landscapes is available [17,18,19], reviewed in [20].

Termite population ecology is classically reviewed by Bignell and Eggleton [7] and by Lepage and Darlington [4], from which it is estimated that live biomass across all termite habitats ranges up to 11 g m^−2^ and numerical density up to 10,000 individuals m^−2^, with 510 to 1150 g of live weight in the largest nests, but there are great differences between habitats and the data in these two reviews have now been updated in many instances. In general, termite species’ richness and abundance declines with decreasing rainfall and increasing altitude, but even with these restrictions landscape-level calculations suggest that termites are dominant soil invertebrates across much of the tropics and subtropics. A classic study of insects in Amazonian rainforest and land-uses derived therefrom by Fittkau and Klinge [21] is often used to support the claim that termites represent 80% of insect biomass and 30% of all animal biomass in those habitats, and that combined with ants account for 95% of insect live weight. Recent claims are more modest. In the natural environment (lowland rainforest) of Sumatra, termites typically comprise 79% of all macroarthropod individuals and 62% of all macroarthropod biomass, with earthworms scarcely represented, but disturbance by logging and conversion to plantation or crop fields reduces the relative representations in favour of ants and (temporarily) greatly in favour of earthworms, especially in semi-natural secondary woodland seeded with rubber trees [8]. Such sensitivities underline the importance of termites, since the soil-feeding species with high abundance and biomass suffer the greatest declines when forest habitats are disturbed, and their removal may diminish the ecosystem services for which they are principally responsible. However, there is also some evidence to support the intermediate disturbance hypothesis [7]. Even the most resilient forest termites cannot recover from complete tree clearances; consequently, the conversion of natural woodlands to other uses often begins a degenerative sequence in which biodiversity, soil fertility, soil ecosystem resilience and soil physical stability all decline. In natural savannas there are different dynamics, as most termite species are strongly adapted to moisture conservation and land use changes tend to manifest themselves in the reduction of woody resources on which termites feed. Evidence of intercontinental variations in typical termite abundances and biomasses is presented by [22]. An early review of termite and earthworm impacts on ecosystems by Lavelle et al. [14] is still useful and very widely cited.

Recent work has pointed to the important role of termite mound and runway building in pedogenesis and nutrient recycling. The constructions involve particle selection, generally favouring clays, and may also concentrate organic matter and some nutrients [12,23,24]. The larger mounds may consequently support a separate community of plants and function as point-scale biodiversity refugia [12,25,26]. However, this is not inevitably the case and parent soil type is still reflected in the constructions [23]. Field exclusion experiments (not using insecticides) have recently resumed following a period of abeyance when they were considered impractical for soil organisms and show that the presence of termites, with some other insects, accelerates woody decomposition [27]. Litterbags accessible to termites show that there is a preference for larger sized woody items, at least in warm temperate forests, a conclusion also reached by [28] using a line intersection sampling approach.

Greenhouse gas emissions by termites attract attention periodically. Measurements averaged across a wider range of species and feeding groups than those addressed in early work have suggested that termites mediate about 2% of the CO_2_ flux to the atmosphere from all terrestrial sources, but this apparently low figure may conceal the physical processing (comminution) of up to 30% of net primary production in some habitats [8,29]. The few detailed studies at the landscape level also suggest significant contributions, for example Konaté et al. [30] estimated that in the Guinea savanna of West Africa, CO_2_ emissions by termites represented 4.9% of aboveground net primary production and 11.3% of the carbon not mineralised by annual fires. An earlier study in a similar savanna by [31] had concluded that termites were responsible for about 20% of total carbon mineralisation (roughly the same as mammalian herbivores and bush fires, respectively), while Yamada et al. [32] estimated that 11.2% of aboveground wood and leaf litter was mineralised by termites in the dry evergreen forest of Thailand. While these figures are impressive for a single invertebrate taxon below ordinal level, there are clearly great variations between habitats; in tropical forests where termites are especially abundant and may consume a third of leaf litter, termite CO_2_ emissions are small compared to tree root respiration, whereas in arid savannas and deserts where termites have lower overall biomass, their share of C mineralisation from wood and grass may nevertheless be as high as 90% [8]. Global methane emissions to the atmosphere from termites are now estimated to be no more than 4%–5% of all sources and may be much less because methane has been shown to be oxidised in mound materials and the soil adjacent to nests [29,33,34].

## 5. Re-Defining Termite Ecology

Table 2 suggests the following:

- Discussion of sampling methods for species diversity in natural or man-made ecosystems has continued but confirms the basic superiority (for time-limited excursions), of transect-based surveys over randomised quadrats for basic information on functional group balances. Progress with sampling in savannas is also notable.
- The existing consensus on the definitions of feeding groups remains largely, though not universally, accepted.
- There has been a large increase in articles concerned with epigeal termite mounds: their mineral (i.e., mineral particle and nutrient) properties and historical turnovers, together with those of associated gallery sheetings.
- The role of termite mounds and spacing in landscapes (especially with respect to colonisation by plants and consequent interactions with mammalian herbivores) continues to be investigated, especially in savannas.
- Genomic methods of identification of specimens, and subsequent phylogenetic reconstructions of local assemblages, can now be considered standard procedure for field work.
- Much biogeographical data on termite diversity and abundance remains to be collected and logged at point, plot and regional scales.
- The role of termites in ecosystems, especially that of their definitive contributions to organic decomposition, detritus shredding and soil conditioning has received little experimental (as opposed to rhetorical) investigation. In other words, that more exclusion experiments are still a priority, within the regulations for insecticide use, but can perhaps be circumvented by litterbag or line-intersection methodologies.
- Predation by ants may be a significant factor in termite population ecology.
- By common consent, termite biodiversity appears to matter to ecosystem stability, but definitive evidence is still in short supply.
- Invasion and climate change are new features in the discussion of termite ecology.
- Isotope analyses have the potential to better define termite feeding niches.

## 6. Additional Review Literature

More than 40 articles published since 2000 can be considered as relevant review material, in whole or in part. The following 13 may be of particular interest. These cover termite ecology [35]; global biogeography [36]; ecosystem services and impacts [37,38,39,40]; termite/vegetation dynamics in savannas [41,42]; subfamily or genus focus [43,44]; invasive termites [45]; use of stable isotope ratios [46] and metabolic scaling to niche [47]. Note that this listing is not inclusive and much additional literature will be identified elsewhere in this volume. A late addition to the literature [48] provides evidence that termite activity is enhanced under periodic drought conditions in tropical rain forests, thus revealing their likely role in stabilizing ecosystems under climatic stress.

## 7. Conclusions

Termite ecology in the 21st century remains a buoyant field, with more than 400 articles published since 2000 which have obvious or arguable relevance. Fieldwork in natural and semi-natural ecosystems now follows more standardised procedures based on transects and there has been a large increase in diversity and population data from dry tropical forests and from savannas. Concerning the impacts of termites, there is a focus on the bioturbation and nutrient cycling that result from their constructions and while it is accepted that termites are, in most cases, beneficial to soil health and ecosystem heterogeneity, there is little definitive evidence and devising effective exclusion experiments remains a challenge.

## Figures and Tables

**Table 1:** Number of articles published in each year between 2000 and 2018 (inclusive) retrieved from Web of Science^TM^ (Thomson Reuters) using termite + one other term (including alternates) as keywords in a topic search, shown in decreasing numerical order. Searched 18 February 2019. Note that any single article may be included in more than one category.

Keywords	Number of Articles 2000–2018
Termite	6722
Termite + bacteria or fungus or protist or protozoan or flagellate	1650
Termite + control	1228
Termite + behaviour	1101
Termite + fungus	958
Termite + evolution	780
Termite + bacteria	687
Termite + lignocellulose or lignin or cellulose	605
Termite + ecology	436
Termite + genomic or proteomic or metagenomic or transcriptomic	182
Termite + invasion	78
Termite + sociality	69
Termite + climate change	58

**Table 2:** How the literature on termite ecology has expanded and diversified in the 21st century. The table lists the headings and sub-headings in Bignell and Eggleton 2000 [7], together with the number of citations (round brackets) included under both the main text headings and subheadings, and in addition the new subheadings and citation totals that are now required to develop the scope of the Millennial review. Note that the same citation can appear in both the first and second columns. New main headings are identified in the last column. For articles published after 2000 (including those with a previous publication date, but now considered significant without being included in [7]), the number tabulated corresponds to the listing of the article on Web of Science^TM^, i.e., one listing earns one citation however many times the article is listed in subsequent published bibliographies. Therefore, the same citation appears in only one of columns three to five.

Main Text Headings in Bignell and Eggleton 2000 [7]	Sub-Headings in Bignell and Eggleton 2000 [7]	Number of New Articles Included in Existing Sub-Headings	Additions to Existing Sub-Headings, 2000–2018	New Main Headings Needed in 2018
INTRODUCTION AND EXISTING LITERATURE (51)	-	-	-	REVIEWS OF TERMITE ECOLOGY (42) **
HOW IS A TERMITE ASSEMBLAGE STUDIED? (1)	Species richness sampling (16)	15	-	ADDITIONAL APPROACHES (3)
	Sampling abundance and biomass (44)	13	Sampling mound density at the landscape level (4)	[Rearing soil-feeders in the lab] (1)
	Estimates of attack on substrates and consumption of food (30)	4	Exclusion and mulching experiments (11)	[Arena interactions and food selection trials] (2)
			Analyses of stable isotopes (5)	-
THE ELEMENTS OF A TERMITE ASSEMBLAGE (71)	Feeding groups (62)	9	Gallery and mound properties, mound history, colony size and secondary occupants * (24)	-
	Nesting structure, gallery sheeting and mound properties, mound history, colony size and secondary occupants * (8)	24	Soil particle selection (4)	-
			Properties of termite sheetings (4)	-
			Genetic and phylogenetic structures at field level (3)	-
CURRENT INFORMATION ON TERMITE ASSEMBLAGES (4)	Data from natural ecosystems (94)	60	Cryptic species (4)	-
			Soil types, host plant and termite microscale distributions (10)	-
	Changes along natural and anthropogenic gradients (13)	46	Termites and climate change (3)	-
ROLES OF TERMITES IN ECOSYSTEM FUNCTIONING (17)	Selection and consumption of food (25)	31	Predation on termites (6)	-
	Energy fluxes (7)	5	Contributions to organic decomposition (18)	
	Does biodiversity matter? (28)	1	Ecosystem services and soil health (28)	
			Distribution of plants, herbivores, crop pests (23)	
			Conservation and restoration of landscapes (14)	
Numerical total (144)	327	208	161	48

* Amendment to the existing sub-heading; ** All citations listed here as reviews are given in full in the bibliography.
